# Quantifying habitat selection and variability in habitat suitability for juvenile white sharks

**DOI:** 10.1371/journal.pone.0214642

**Published:** 2019-05-08

**Authors:** Connor F. White, Kady Lyons, Salvador J. Jorgensen, John O'Sullivan, Chuck Winkler, Kevin C. Weng, Christopher G. Lowe

**Affiliations:** 1 Department of Biological Sciences, California State University Long Beach, Long Beach, California, United States of America; 2 Monterey Bay Aquarium, Monterey, California, United States of America; 3 Aquatic Research Consultants, San Pedro, California, United States of America; 4 Virginia Institute of Marine Science, College of William & Mary, Gloucester Point, Virginia, United States of America; Department of Agriculture and Water Resources, AUSTRALIA

## Abstract

While adult white sharks (*Carcharodon carcharias*) are apex predators with a circumglobal distribution, juvenile white sharks (JWS) feed primarily on bottom dwelling fishes and tend to be coastally associated. Despite the assumedly easier access to juveniles compared to large, migratory adults, limited information is available on the movements, environments, and distributions of individuals during this life stage. To quantify movement and understand their distribution in the southern California Bight, JWS were captured and fitted with dorsal fin-mounted satellite transmitters (SPOT tags; n = 18). Nine individuals crossed the U.S. border into Baja California, Mexico. Individuals used shallow habitats (134.96 ± 191.1 m) close to shore (7.16 ± 5.65 km). A generalized linear model with a binomial distribution was used to predict the presence of individuals based on several environmental predictors from these areas. Juveniles were found to select shallow habitats (< 1000 m deep) close to land (< 30 km of the shoreline) in waters ranging from 14 to 24°C. Southern California was found to be suitable eight months of the year, while coastal habitats in Baja California were suitable year-round. The model predicted seasonal movement with sharks moving from southern California to Baja California during winter. Additionally, habitat distribution changed inter-annually with sharks having a more northerly distribution during years with a higher Pacific Decadal Oscillation index, suggesting sharks may forego their annual fall migrations to Baja California, Mexico, during El Niño years. Model predictions aligned with fishery-dependent catch data, with a greater number of sharks being captured during periods and/or areas of increased habitat suitability. Thus, habitat models could be useful for predicting the presence of JWS in other areas, and can be used as a tool for potentially reducing fishery interactions during seasons and locations where there is increased susceptibility of incidental catch.

## Introduction

Individuals are constantly responding to shifts in environmental conditions and vary their distributions accordingly to maximize fitness [1–5]. By understanding the association between animals and their preferred conditions, researchers can use this information to model animal movements. Furthermore, with the advancement of computational tools and collection of high-resolution environmental data, spatio-temporal models can be developed to examine and predict how animals interact with their environments. This has implications for how we manage direct and indirect human-animal interactions, such as exposure to fishing activity, marine pollution and shipping traffic across spatial and temporal scales [6–11].

However, physiological tolerances to environmental conditions, predation risk, and prey availability, vary across ontogeny and result in juveniles of some species selecting different habitats than adults [12–14]. Understanding the environmental drivers of juvenile distributions is particularly vital as many species exhibit naturally high mortality during the juvenile stage, which is often one of the strongest limitations to population growth [15–19]. However, identifying the spatio-temporal location of suitable nursery habitat for species with low natural densities and high mobility is challenging, representing an impediment to conservation efforts by hampering population recovery of overexploited species.

The white shark (*Carcharodon carcharias*) exemplifies a species that exhibits both ontogenetic shifts in habitat use, diet and thermal physiology and is listed as vulnerable by the International Union for Conservation of Nature (IUCN) [20]. While subadult and adult white sharks are known to aggregate around marine mammal colonies, neonatal (<150 cm total length-TL) and young-of-the-year (YOY, <175 cm TL) white sharks are not found in these aggregations. These juvenile sharks have been most commonly observed or captured in nearshore, temperate waters during the warmer months of the year, where their diet largely consists of smaller benthic elasmobranchs, teleosts, and invertebrates [21–24]. Factors driving the separation of adult and juvenile habitat are not well understood, but may be related to differences in diet or physiology between adults and juveniles.

Understanding factors that drive juvenile habitat selection and at what times of the year sharks are likely to be present is important for accurately determining annual recruitment, population trajectory and effective conservation of white sharks. Furthermore, understanding the spatio-temporal overlap between fisheries and nursery areas is vital to understanding the degree to which humans may interact with this species of conservation concern. There is evidence that white shark populations in some parts of the globe may be positively responding to previously implemented conservation measures [22,24], however, there is little historic data on white shark nursery use or population sizes. Predicting and identifying what habitats could be utilized by this species as populations grow is necessary for understanding population recovery.

In order to determine how juvenile white sharks (JWS) select habitats, quantifying the oceanographic conditions and key habitat features that influence the presences of individuals is necessary for developing informative models. However, due to their low natural densities, relying on observations of catch data alone is insufficient to quantify the locations of individuals. By satellite tagging individuals and overlaying their movements onto the actual oceanographic sea state, we aimed to define the environmental conditions (depth, temperature, primary productivity) used by juvenile sharks. Using these characteristics, from tracks spanning months, we were able to predict the spatio-temporal patterns of suitable habitat for juveniles in the Northeast Pacific. To test the accuracy of the model, we compared commercial gillnet fishing bycatch time and locations to demonstrate their utility in predicting fishery interactions and how it can be used as a future management tool. To examine the global applicability of JWS nursery habitats, we extrapolated this model across all coastal areas worldwide.

## Methods

### Capture and tagging

From 2006 to 2009, in collaboration with local commercial gillnet fishers, incidentally captured JWSs were brought to the nearest port in a large fish tote (1.2 x 1.2 x 1.2 m) with flowing seawater. Researchers then met fishers to physically assess, measure, and tag sharks. JWSs were also captured directly using hook-and-line or small commercial purse seine, where they were brought onboard, measured, and tagged immediately. Incidentally caught sharks were released 2 km offshore from the port of assessment, while targeted individuals were released at the site of capture. A subset of these directly targeted individuals were transported to the Monterey Bay Aquarium for public display for 11–138 days, and then subsequently tagged and released off Monterey, California (for information on captive handing, display and post release behavior see[25–27]). After release, these individuals displayed behavior that was consistent with individuals released immediately after capture, thus data were treated the same as individuals released after capture.

All sharks were fitted with Smart Position Only Tags (SPOT, mini SPOT 5AM-S182C and AM-S183E, Wildlife Computers) that were attached to their dorsal fins using nylon bolts [28]. Additionally, a subset of individuals opportunistically received a popup satellite archival transmitter (PAT, Mk10-PAT, Wildlife Computers), however, this data was not used for estimating habitat selection. All tagging work was conducted under California Department of Fish and Wildlife (CDFW) permit (#3450) and all procedures on animals were reviewed and approved by the California State University Long Beach Institutional Animal Care and Use Committee (IACUC) under permit number 274.

### Location estimation

Geolocations of tagged sharks can only be generated when the fin-mounted tag breaks the water’s surface and an orbiting ARGOS satellite is overhead. The accuracy (<250 m to >10 km radius) and frequency (hourly to weekly) of SPOT locations is highly variable, depending on satellite coverage, individuals’ surface-oriented behaviors, the number of transmissions, sea state, and the time between transmissions [29]. These sources of variability in transmission data can impart certain biases in interpretation of movements. Therefore, only the highest quality SPOT tag locations (Argos class 1–3) were used and standardized into mean daily positions for each individual to reduce pseudo-replication. We used this approach to reduce the potential bias of some individuals having multiple transmissions on a given day, to make day, not individual transmission, the statistical unit.

Many studies interpolate positions to standardize for differential spatial and temporal detection probability; this produces tracks of positions that are equally-spaced throughout the study. However, interpolating locations would increase the statistical sample size by creating positions for days in which no transmissions were received, thus increasing the resultant power of statistical analysis. Additionally, if there was a spatial bias in detection ability, interpolating between these positions could reinforce, rather than eliminate this bias, by placing interpolated positions in areas of high detection probability. Additionally, as we were examining habitat selection on small spatial scales <4km, interpolations of positions could introduce additional error. Thus, based on the observed JWS tracks and the scale of habitat selection we were interested in, locations were not interpolated for days in which there were no received transmissions, to best reduce potential bias. See S1 and S2 Figs for comparison of habitat selection differences between daily standardized and interpolated datasets.

### Habitat selection

To determine shark habitat selection preferences, environmental data for each shark location were extracted. Environmental parameters included water depth, distance to shore, sea surface temperature (SST), SST gradient, Chlorophyll A, and Chlorophyll A gradient at surface detection location (see S1 File). These parameters were chosen as temporal and spatial data were available at fine resolutions (< 1 week and < 4 km, respectively) for the entire globe, and closely matched SPOT tag geolocation resolutions to minimize spatio-temporal mismatch between the environmental and SPOT location data.

A generalized linear model (GLM) with a binomial distribution was used to compare the habitats used by tagged animals compared to available habitat. Available habitat was determined for each detection by using a diffusion model with the probability of a location being available for each individual shark dependent on the distance to its release location and the time since release (Eq. 1). The rate (diffusion constant) at which habitat becomes available was calculated as the mean square displacement from observed SPOT tracks.

$$P(x,t)=\frac{1}{\sqrt{4\pi Dt}}e^{\frac{({x-x_{0})}^{2}}{4Dt}}$$ (1)

Using this model, pseudo absences were set as randomly selected locations based on their probability that the location would be available to the shark. Randomly selected points were chosen in this fashion as this procedure yields more reliable results when using regression-based techniques [30]. A large number of random points (n = 1000) were collected at each time point to pair with each presence (detection) point. Using this approach, the pseudo absences do not reflect where the shark was not, but an approximation of where the shark might have been. In the regression, pseudo absences were given a weight of 1/1000 so that the weighted sum of presences equals the weighted sum of pseudo-absences [30].

### Model selection

A full complement of models was constructed with the serial removal and addition of each environmental parameter. Quadratic terms were also included for SST and chlorophyll to determine if individuals were only selecting for certain ranges of these parameters, as many organisms exhibit upper and lower selection criteria. However, in order to improve model interpretation, biological relevance and limit the over parameterization of models, no interaction terms were included. All models were compared to a null model with no predictors, suggesting that individuals were not selecting any habitat features. The models with the greatest change in the Akaike Information Criterion (AIC) from the null model that did not differ by at least 2 AIC points were considered candidate models, with the selected model as the one with the fewest number of parameters [31].

### Suitable habitat identification

Habitat suitability was defined as the probability of a habitat being used based on the coefficients from the GLM (Eq 2).

$$P(Selection)=\frac{1}{1+e^{intercept+c_{1}*{Env}_{1}+c_{2}*{Env}_{2}+\cdots }}$$ (2)

Daily habitat suitability maps were constructed for the entire study area from 2002–2015 by calculating the habitat suitability of each cell in the study area based on corresponding environmental maps. Core habitat was identified as the cells that contained the top 25 percent of the sum of the habitat suitability. As the coastline along the Southern Californian Bight (SCB) and Baja California, Mexico, is largely aligned North-South, latitude was used as a proxy of the location of suitable habitat. The median latitude of the 25 percent core areas was calculated each month throughout the model period. To test for seasonal differences in the distribution of suitable habitat, a linear mixed model with a random effect of year was used to determine if median latitude across each season significantly differed. To determine if oceanographic regime shifts affect the distribution of predicted suitable JWS habitat, the mean position of white shark habitat during each month was regressed against the Pacific Decadal Oscillation (PDO) index [32].

### Model verification and application

To verify the model predictions of suitable habitat, incidental JWS capture records were spatially and temporally compared with model predictions. Total gillnet fishing effort (e.g., set duration in hours, length of net in fathoms, target species, etc.) was obtained from gillnet logbooks for the years 2004–2009 for southern California (defined here as the region between Santa Maria and San Diego) reported by CDFW commercial fishing block (10’ X 10’ blocks). Since gillnet length regulations vary among the fisheries, fishing effort data were normalized by dividing net soak hours by fathoms of net length for each fishing block each month (herein “effort” refers to standardized data unless otherwise noted) [33]. Suitabilities were aggregated to be at the same coarser spatio-temporal resolution as CDFW blocks. This was done by averaging the suitability of all locations within a CDFW block each month.

Date and location (i.e. block) of incidental white shark captures were obtained from the CDFW archive of logbook records (2004–2009), where reporting of incidentally captured white sharks is mandatory for commercial gillnet fisheries in southern California. The number of white sharks caught in each block was regressed against both predicted suitability and standardized effort to determine if predicted suitability correlated with JWS bycatch. This was done using a generalized linear mixed model from the lme4 package in R [34] with the number of individuals being specified as a Poisson distribution. Fishing block was classified as a random variable to account for the non-independence in the dataset.

### Global habitat identification

Based on the habitat use of tagged sharks in the SCB and verification from fishery comparisons, habitat suitability for JWSs was projected globally. This was done by extracting daily global environmental variables from Jan 2013—Dec 2015, and generating monthly predicted suitability maps using Eq 3. Potential areas suitable for JWS were defined as the smallest area that contained 50 percent of the total probability of presence based on environmental conditions found for SCB JWS and independent of historic/actual white shark observations for a given area. For areas that were available to individuals for a substantial portion of the year, maps were constructed showing the number of months each area was considered suitable habitat.

## Results

### Tagging

From 2006–2009 a total of 18 sharks were outfitted with SPOT tags (S1 Table). Fourteen individuals were tagged as a result of incidental gillnet capture, while four individuals were tagged from directed fishing efforts (hook-and-line: n = 1, purse seine: n = 3). All individuals were captured between San Diego and Santa Barbara, California, and released close to the site of capture, except for two individuals that were released in Monterey Bay after being kept on exhibit at the Monterey Bay Aquarium. Of the eighteen, one individual died immediately after release as indicated by its PAT tag, and recorded no locations (JWS_08–18), another individual was likely eaten 36 days after release (JWS_08–07) and six individuals were subsequently captured and died in gillnets after release (JWS_06–13: 21 days, JWS_ 08–01: 233 days, JWS_ 08–02: 614 days, JWS_ 08–13: 124 days, JWS_09–11: 113 days, JWS_09–12: 14 days).

There was wide variability in the number of geopositions rendered among individuals (Class 0–3: 645 locations; mean per individual: 36, range: 0–130), with most tags reporting less than one year after release (mean ± SD: 107 ± 132 days). The average time between subsequent detections was 3.69 days, and when excluding the three longest intervals (>100 days) the average interval between subsequent detections was 1.99 days. When using the daily standardized locations, 15% of subsequent locations were after more than 5 days. Distances traveled between subsequent detections were small (31 km) and 50% of subsequent locations were within 15 km of their previous location even after 5 days. As such, these tracks poorly reflect the true path that the animal traveled. However, all individuals did not remain in the same area throughout the entire study, with nine individuals indicating movements south of the U.S. border to Baja California, Mexico, between the months of August and January (S3 Fig). During these times individuals displayed directed movements with average displacement speeds up to 1.5 km/h.

### Habitat selection

**Fig 1:**
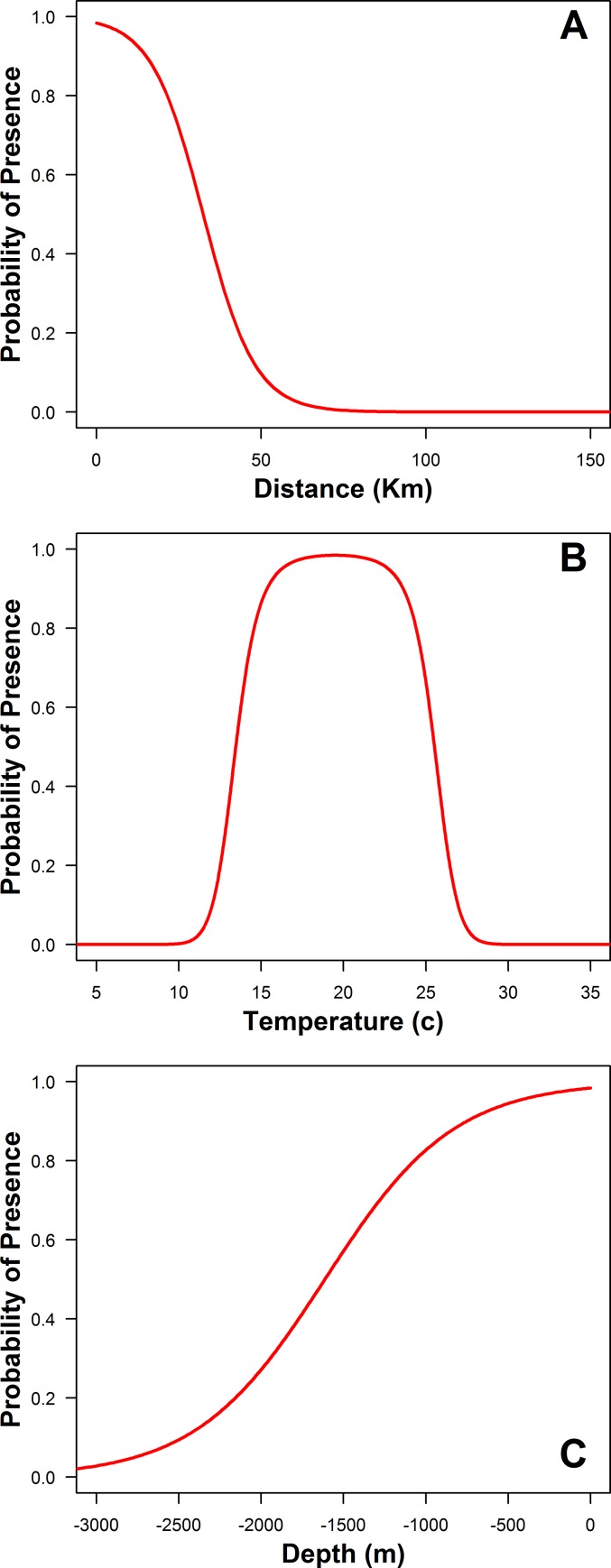
Habitat suitability by environmental parameters. Presences (standardized daily positions) are shown as black circles with a value of 1, while available points are shown as a having a value of 0. Values are plotted against distance to land (A), temperature (B) and depth (C). Conditional predicted responses for each variable are displayed as red lines by fixing the other two parameters.

Sharks were found in surface waters between 14.1°C and 26.3°C (mean ± SD: 18.61 ± 2.25°C) over depths ranging from <1 to 2188 m (134.96 ± 191.1 m) and in habitats <1 to 59.8 km from the shoreline (7.16 ± 5.65 km). An individual’s presence was significantly predicted by depth, distance to land, temperature, and the temperature quadratic term, indicating sharks are selecting for temperatures between an upper and lower limit (ΔAIC = 748.1859, GLM *Depth*: Z = 4.591, p <0.001, *Distance*: Z = -6.067, p < 0.001, *Temp*: Z = 5.255, p <0.001, *Temp*^*2*^: Z = -5.181, p <0.001). Individuals selected for areas that were close to land (<30 km) with water depths shallower than 1000 m and water temperatures between 14 and 24°C (median 19°C) (Fig 1). Based on the coefficients derived from the habitat selection function, habitat suitability was calculated using Eq 3. Depth gradient, temperature gradient, chlorophyll, and chlorophyll gradient were all found to not significantly improve model fit and were not used in predicting habitat suitability.

$$P(Habitat)=\frac{1}{1+e^{-(-27.798274+0.002244*Depth-0.000103*Distance+3.460984*Temp-0.09243*{Temp}^{2}}}$$ (3)

### Suitable habitat distribution

**Fig 2:**
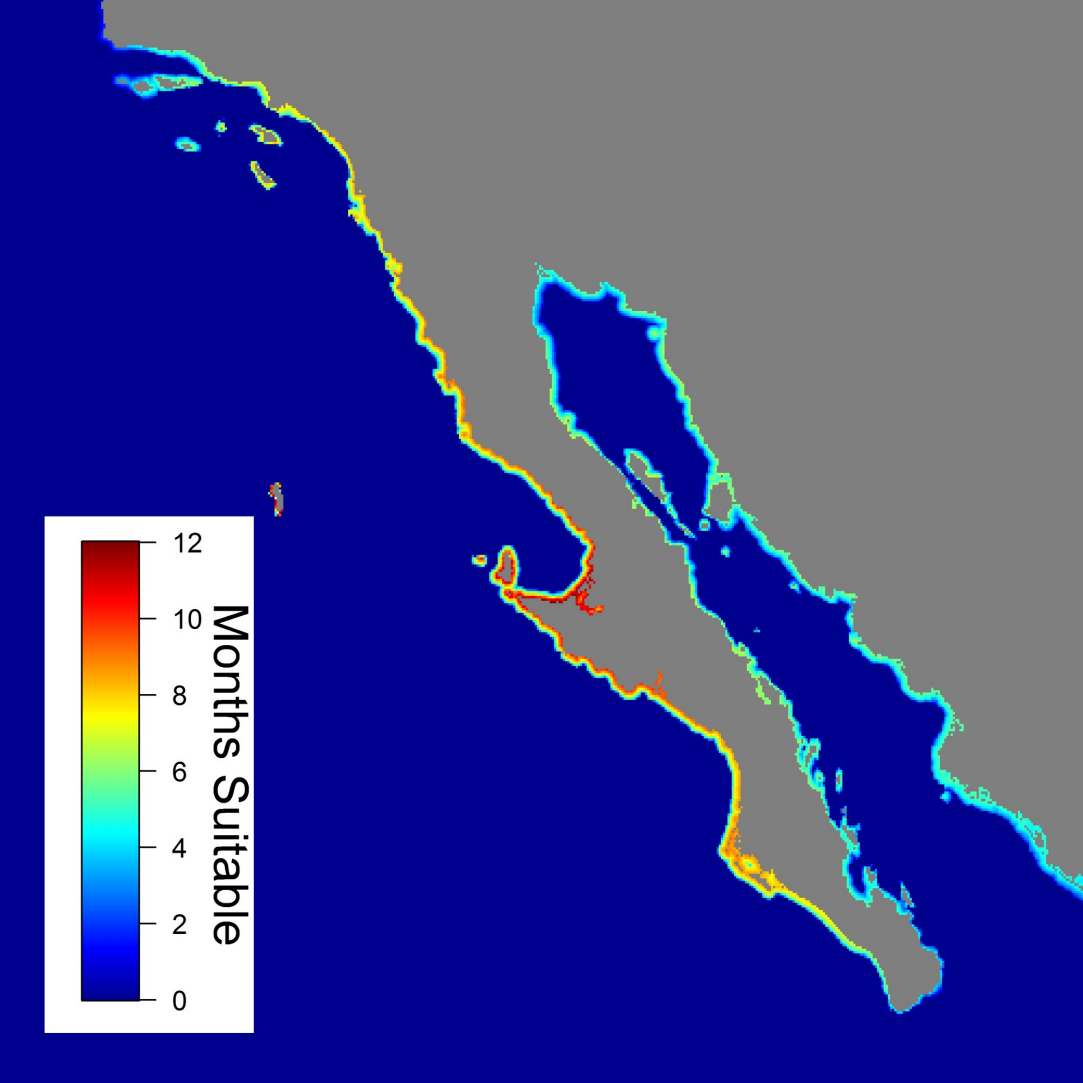
Map of habitat suitability in the Northeast Pacific Ocean. Map of the Northeast Pacific Ocean, with colors representing the monthly overlap in the 25% core area.

**Fig 3:**
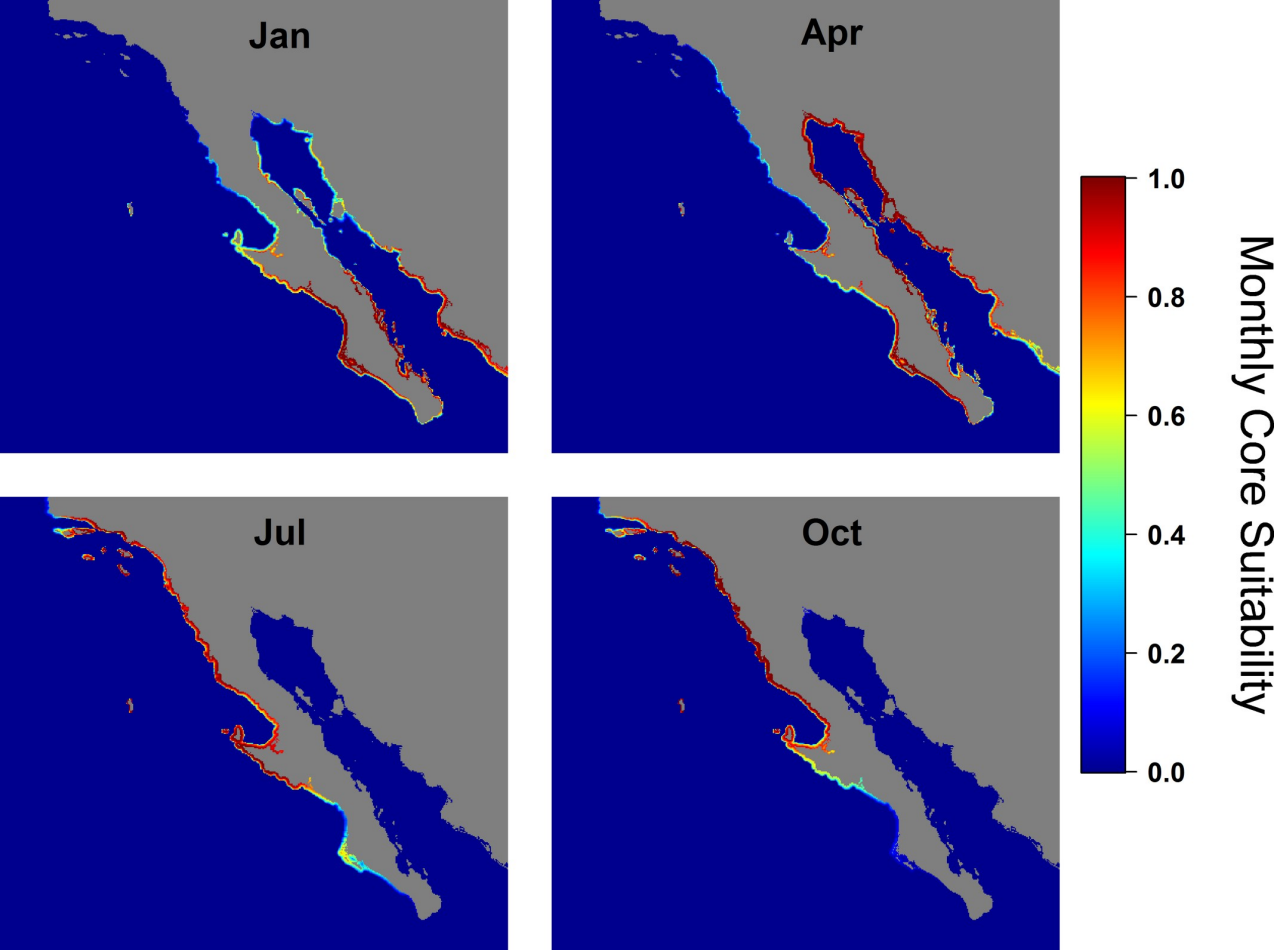
Monthly habitat suitability in the Northeast Pacific Ocean. Areas of suitable habitat for JWS in the Northeast Pacific by each month of the year, with red being areas that are highly suitable while blue is unsuitable. Monthly averages are based on environmental variables throughout the study period (2002–2015).

**Fig 4:**
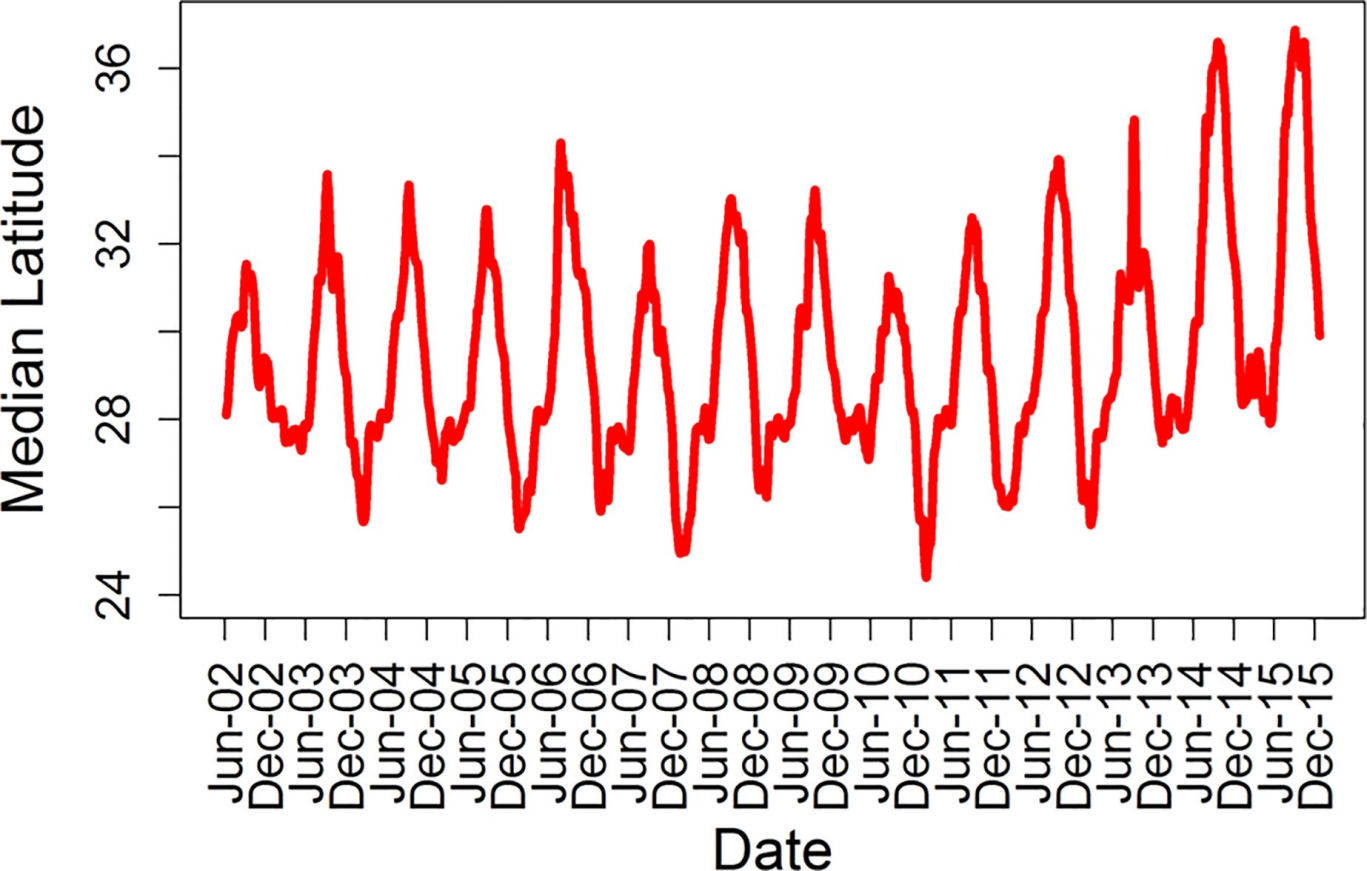
Median suitable latitude over time. The 10-day running mean of the median latitude of the 25% core area used each day. There were significant monthly and yearly differences in the median position. In the summer of 2014 and 2015 there were northerly expanses in median core latitude.

Based on individuals’ habitat selection, suitable habitat distributions were extrapolated for the entire Northeast Pacific, with the most suitable locations (defined as locations that summed to 50% of the total probability) for each month deemed as core habitat. Year-round JWS core habitat was not available in most areas and inter-annual distribution of suitable habitat was highly variable, with the exception of Vizcaino Bay, Baja California, Mexico, which was suitable all months of the year (Fig 2). Seasonally, there were significant shifts in the median latitude of suitable habitat (Fig 3, mixed model, F_11,4922_ = 2493, p <0.001). Mean position of suitable habitat was significantly and positively related to the PDO index (Fig 4, PDO: F_1,4934_ = 1353, p <0.001). In non-El Niño years, southern California was relatively unsuitable from December through April, while the Gulf of California and much of the Pacific Coast of Baja California was suitable habitat for JWS during these months. During years when the PDO was indicative of El Niño conditions, there was a more northerly shift in suitable habitat, with southern California remaining suitable for JWS during the winter months.

### Model verification and application

**Fig 5:**
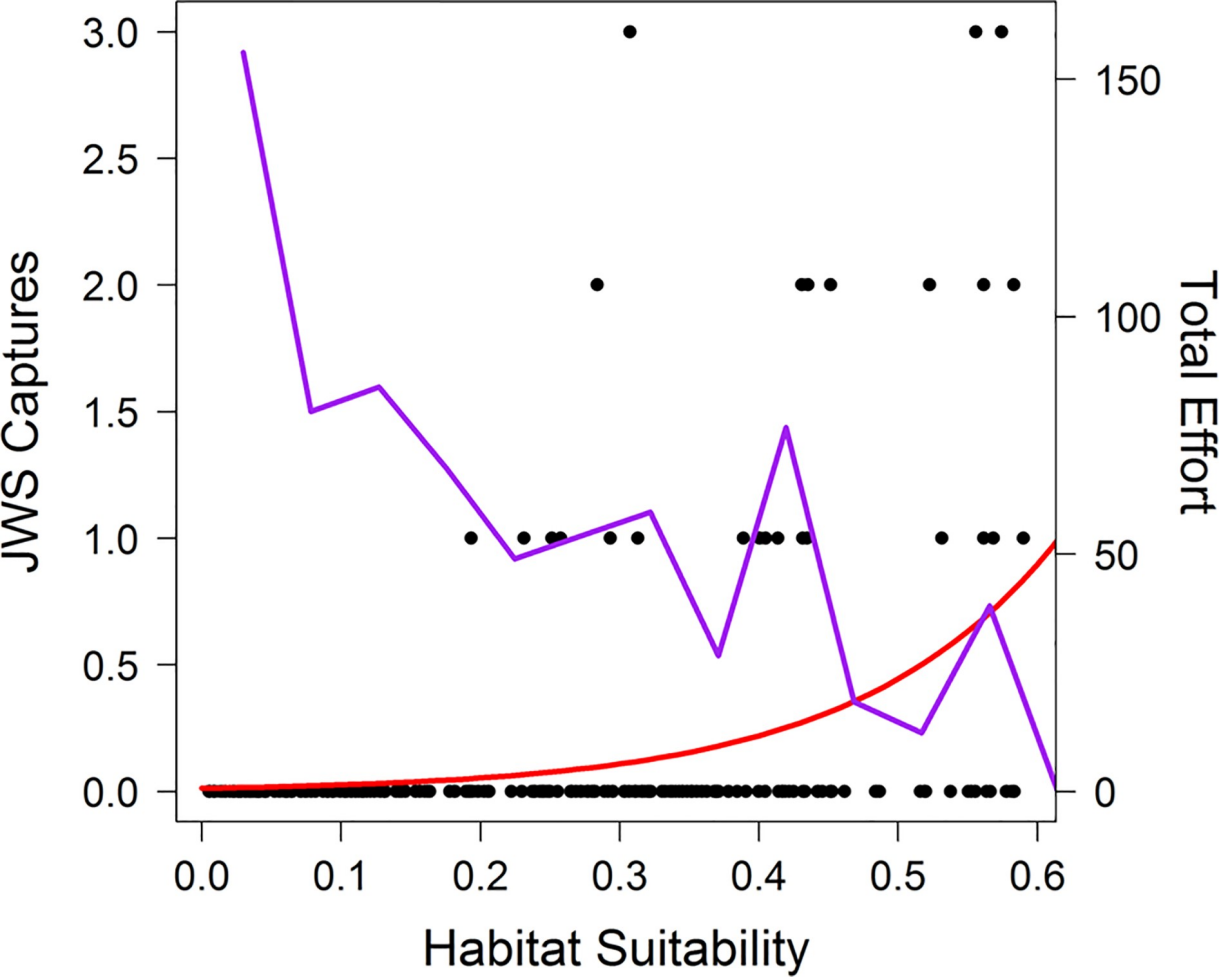
JWS captures by predicted suitability. The number of JWS captured (black circles) in each block-month (left y- axis) against the predicted habitat suitability. Habitat suitability predicted the mean number of JWS caught (red line). Sum total effort (right y-axis, Hours/fathom length) across habitat suitability (purple line) is overlaid, and decreases with increasing habitat suitability.

From 2004–2008, 42 sharks were reported captured in the California gillnet fishery, with a maximum of three individuals being captured in one block during a single month (n = 3). All captures occurred in only eight out of the available 196 blocks (4%) where fishing occurred. As gillnet fishing is not allowed within California state waters (<5.5 km of land), all reported captures occurred outside of state waters (>5.5 km). JWS bycatch was reported in blocks that ranged in suitability from 0.19 to 0.55 out of 1. Six JWS were captured in three of the eight most suitable block-months, with 13 individuals captured in seven of the most suitable 40 block months. Habitat suitability significantly predicted JWS capture; however, fishing effort did not predict capture (Fig 5, GLM: *Suitability*: Z = 6.083, p < 0.001, *Effort*: Z = 0.277, p = 0.781). In addition, catches within a block were highest when the block-months suitability was closest to the blocks maximum suitability.

### Global habitat distribution

**Fig 6:**
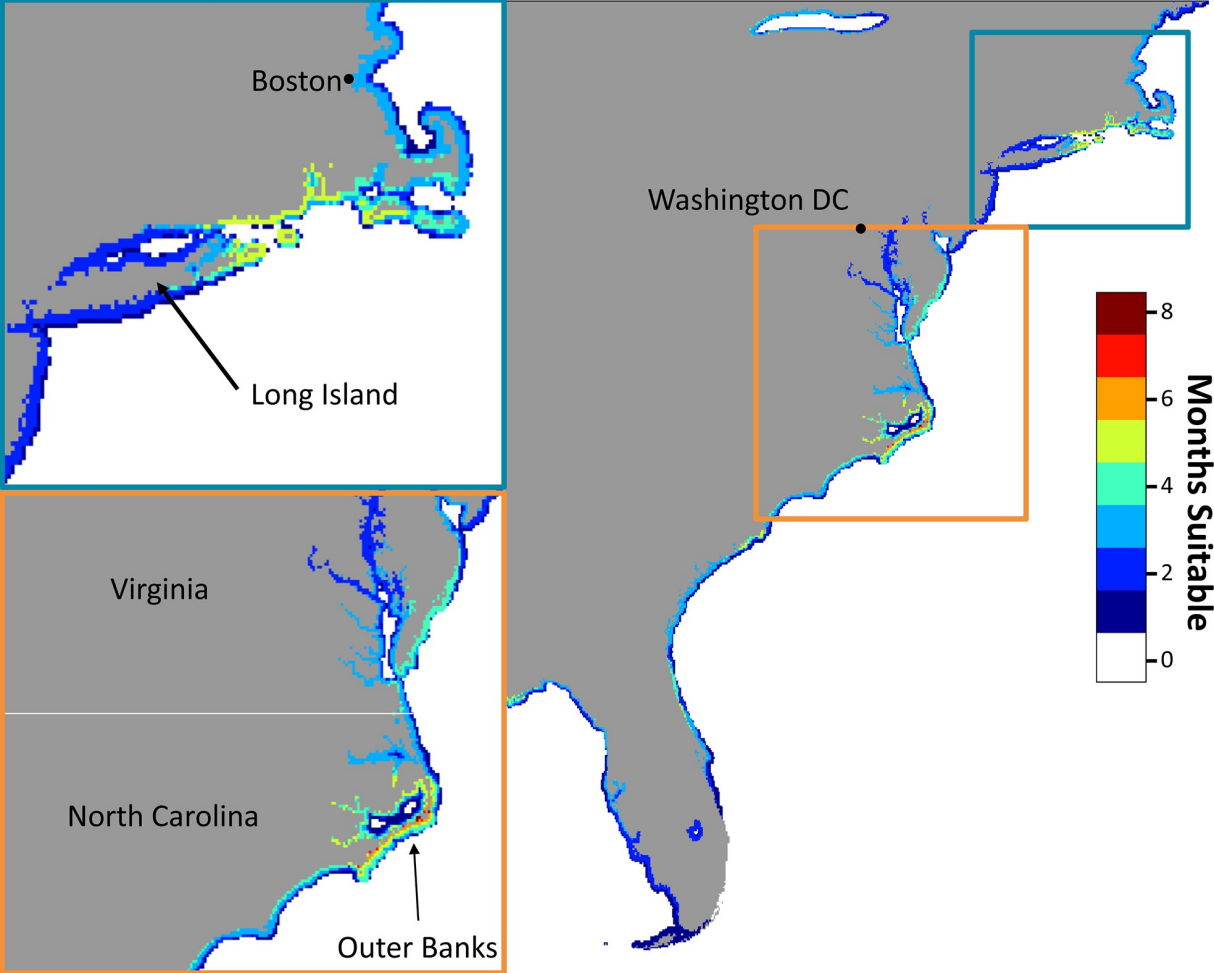
Habitat suitability hot spots for the Northwest Atlantic. Overlap of the monthly 25% core areas for JWS in the Northwest Atlantic. Colors represent the number of months that area represents core suitable habitat for JWSs. There are hot spots off Long Island Sound and off the Outer Banks of North Carolina (insets).

When habitat suitability was calculated globally, seven areas of coastline displayed high degrees of habitat suitability: the Northeast Pacific, Northwest Pacific, Northwest Atlantic, Southwest Atlantic, the Mediterranean/North Africa, South Africa, and Australia/New Zealand. Within each of these coastlines, there were hotspots with suitable habitat available for more than six months of the year. For example, along the Northwest Atlantic, there were two large areas of high potential habitat suitability for JWS, including areas off Cape Hatteras, North Carolina, which could be suitable for up to eight months of the year and Long Island Sound, New York, which may provide suitable habitat for six months of the year (Fig 6).

## Discussion

### Key habitat variables

JWS in the SCB selected habitats based on specific criteria of the variables evaluated. Warm temperate water temperatures, shallow depths and short distance to shore were all found to be important components of preferred JWS habitat. These findings are supported by previous observations of JWS behavior locally and globally [21,23,28,35,36]. Individuals in this study remained predominantly in coastal waters with few individuals making offshore movements, supporting other findings that JWS are coastally associated. This coastal, shallow water association may be related to prey availability as nearshore habitats offer potentially more abundant and easy to capture prey species than offshore, deeper habitats. Stomach contents and isotopic signatures suggest a large component of the JWS diet is composed of other nearshore elasmobranchs and coastal teleosts with little pelagic influence [37]. Additionally, these habitats might provide protection from predators as the only JWS in this study that made an offshore movement was consumed by an unknown predator [38].

Temperature is commonly found to be important in influencing species distributions, as found in the current study, by regulating physiological processes of the focal animal (e.g. muscle performance, metabolism, growth rate) [39–44]. Additionally, temperature simultaneously alters the physiology of other organisms and thus drives ecosystem composition. Water temperature was a significant predictor of JWS presence, with sharks demonstrating specific thermal preferences. JWS were most likely to be detected in habitats greater than 19°C, and less likely in waters cooler than 13°C and warmer than 26°C. JWS in other localities demonstrate similar preferences where 63% of sharks in South Africa were captured between 19 and 22°C [21] and in New South Wales where tagged individuals were found to spend 45% of their time in temperatures between 19 and 20°C [23,36], suggesting that this preference is likely conserved across populations. However, it is important to note that our habitat selection model is based on SST alone, and thus does not represent the thermal limits of the species at this age class, as juveniles are known to spend time at depths with water temperatures as cold as 6°C [28]. Additionally, there is some evidence that individuals can survive prolonged exposure to waters cooler than 14°C. For example, two YOY white sharks that were on display in the Monterey Bay Aquarium were released into Monterey Bay during the winter (January and February), where upon release these animals experienced SST <12° C for over two weeks while they made fairly rapid and directed movements southward until they reached areas that had SST >14° C [27]. These directed movements indicate that individuals were not just utilizing the available habitat around them and did not return to the area from which they were originally captured in, but traveled to an area of warmer waters.

As members of the lamnid family, white sharks display regional endothermy [45]; however, when in their ontogeny they develop endothermic capability is unknown. Since juveniles have larger surface area to volume ratios than adults, they likely lack the mass and physiological means to defend a high body core temperature during prolonged cold temperatures (<10° C) [28,36,46]. Our model supports this hypothesis as JWS selected for habitats with warm surface waters and moved seasonally to follow temperature regimes. However, this does not prohibit them from making forays into deeper waters colder than these preferences as long as they have suitable habitat to return to after making these dives, a behavior observed in many organisms that forage below the thermocline [47–51].

Identifying thermal limits of highly migratory species in a three-dimensional environment is challenging. Since the sea surface represents the warmest part of the water column, it follows that when SST approaches individuals’ lower thermal preferences, they must move horizontally to find warmer water. However, at the upper thermal selection limit, individuals may choose to move vertically, as opposed to horizontally, and remain at depth to access deeper cooler water to regulate body temperatures [51–53]. This would be especially problematic in this and other studies that rely solely on SPOT tags, because when the animals are not at the surface there is no information collected on their location. This problem can be overcome by utilizing PAT tags that record depth and temperature; however, geoposition estimates lack adequate spatial resolution relative to the variation in local environmental conditions that can influence an individual’s movements. Identifying upper thermal selection limit is further complicated in that all the sharks tagged in this study were from the northern end of the suitable potential range for JWS determined by this model. YOY white sharks have been found in the summer and fall off Vizcaino Bay, Mexico, and off South Africa in environments warmer than found in this study (> 25°C) [21,54,55].

### Models for management

Using fishery-dependent incidental catch logbook data we were able to evaluate the predictive ability and, therefore, utility of developing a habitat selection model to reduce JWS-fishery interactions. When fishing blocks had higher monthly predicted habitat suitability, they experienced an increase in JWS bycatch, despite prohibition of gillnet fishing in California State waters (<5.5 km of land). Fishing effort did not predict the number of JWS caught, suggesting that in California the time and areas selected by fishers is somewhat decoupled from those selected by JWS. This contrasts with other areas such as Vizcaino Bay that have high habitat suitability and relatively high fishing pressure, where higher fishing mortalities have been recorded [55].

JWS interactions with fisheries may be further reduced in the SCB as gillnets can only be set in areas outside 5.5 km of the California coastline. Restricting gillnets to areas further offshore and in deeper coastal waters limits the degree of overlap with core JWS habitat. Thus, JWS preference for shallow inshore core habitat explains why gillnet fishing in the SCB represents a much lower threat to JWS in comparison to gillnet fisheries in Baja, Mexico which are not as spatially restricted [38]. When gillnets are set for less than 24 hrs, sharks have a higher (>50%) likelihood of surviving capture if they are immediately released [33]. Based on the performance of this model, managers could use SST data to warn fishers where and when they may have the greatest probability to interact with JWS and encourage them to check their nets more frequently to minimize post-release mortality. Strategies that use environmental parameters to determine the overlap of species of concern with fisheries have been used before when managing different tuna stocks as well as estimating albatross and sea turtle bycatch [7,56,57]. These tools provide an essential function for resource managers to maintain effort in sustainable fisheries while maintaining the fisheries below bycatch quotas [6].

### Northeast pacific habitat

Based on sharks’ habitat preferences, areas of suitable habitat for the Northeast Pacific ranged from Pt. Conception, U.S., to Mazatlán, Mexico. However, within this range there were large seasonal and inter-annual variations in the distribution of suitable habitat. During non-El Niño years, the SCB was only predicted to be suitable for individuals in the late summer through fall, with water temperatures becoming too cold in the winter. This reduction in temperature likely drives the distribution of individuals south of the U.S. border into warmer, Mexican waters as observed in nine individuals from this study. These findings are further corroborated by historic fisheries bycatch data in the SCB, which reports fewer JWS caught during the winter months (January–March) [22].

In contrast, southern areas of Mexico, such as the southern tip of Baja and the Gulf of California, were predicted to be unsuitable for JWS from the summer to fall as waters become too warm, but larger individuals have been observed based on both fisheries-dependent and -independent data [27,54,58,59]. In particular, the central Gulf of California has been hypothesized to be a birthing area for JWSs [35]. According to the model output, the areas around Bahia de Los Angeles, and Isla Angel de la Guarda, were the most suitable habitats in the Gulf of California because deep water upwelling cools this area extending the suitability to seven months of the year.

Vizcaino Bay and Bahia San Ignacio were predicted to provide nearly year-round suitable habitat for JWS and these areas are documented nursery habitats, with individuals captured year-round [55]. During summer months, neonatal white sharks (<1 month old) are found in both the SCB and Vizcaino Bay [22,54,55]. Individuals tagged in the northern SCB have migrated to Vizcaino Bay in the winter and returned to southern California the following summer (JWS 09–09 this study, C.G. Lowe et al., unpub. data). However, it is unknown whether YOY white sharks found in Vizcaino Bay in the summer remain there or whether some may migrate into southern California waters. Determining the connectivity between these areas is important for conservation and management. Efforts should focus on tagging individuals in Baja nursery to determine the degree of movement from Mexican into U.S. waters; however, the magnitude of movement would likely depend on the inter-year variation in suitable habitat as sharks are less likely to move from areas when preferred habitat is immediately available.

### El Niño southern oscillation events

During El Niño years, surface waters in the Northeast Pacific can warm to more than 5° C above average [60]. During the strong 2015 El Niño, areas as far north as Monterey Bay were predicted to provide suitable habitat for YOY white sharks. It is extremely rare to find YOY white sharks in this area; however, two one-year-old white sharks that were tagged with acoustic transmitters in southern California were detected in Monterey Bay in September 2014 and several individuals were observed along beaches in Monterey in September 2014 and 2015, (J. O’Sullivan and D. Ebert Pers Comm, C.G. Lowe et al., unpub. data). Furthermore, in the winters of 2013–14 and 2014–2015 southern California experienced increased water temperatures, with winter surface waters rarely going below 14° C. During these winters, JWS tagged with PAT and acoustic transmitters were observed to remain in southern California (C. Lowe et al., unpubl. data). Thus, it appears that some individuals are able to use opportunistically more northerly available habitats during strong El Niño periods. This is a commonly observed pattern with many coastal subtropical species such as smooth hammerhead sharks (*Sphryna zygaena*) experiencing range expansions into California during these cycles [61,62]. Additionally, there was a significant predicted range reduction south of Point Conception with little predicted available habitat in the Pacific waters of Mexico and there were fewer individuals than normal observed in Vizcaino Bay (E. Garcia-Rodriguez, Pers.comm.). The patterns found with El Niño Southern Oscillation events have significant implications for how the availability of suitable JWS will be affected rise ocean temperatures resulting from climate change. Thus, habitat modeling is an important tool managers can use to forecast how species distributions may shift in the coming years. Furthermore, El Niño Southern Oscillation events have been found to affect documented sub-adult and adult white shark predation rates in South Africa [63].

### Global estimates of white shark nursery habitat

While caution is needed when extrapolating findings to regions outside of the study area, especially when there are potential spatial collection biases, predicting the global location of JWS habitats revealed overlapping areas with known JWS captures. The Northwest Atlantic, South African, the Mediterranean and Southeast Australian coastlines have all previously been reported to be potential nursery areas for JWS [21,24,35,36,64]. Despite some of the potential problems with global model extrapolation there was evidence of congruence between model predictions and known nursery areas.

When examining the Northwestern Atlantic, there are areas that display higher suitability over longer periods of time, such as Long Island Sound, which contained the largest catches of YOY on the east coast of the U.S. [24]. Recent JWS tracking results from the North West Atlantic indicated that JWS caught and tagged off New York during the summer spent their first winter months off the outer banks, an area that is highly suitable for JWS during the winter [65]. While model predictions appear to be fairly robust, the areas identified are large, and may pose challenges in confirming JWS presence but provide a baseline for understanding the spatial location of potential nursery areas.

Obviously, there are many factors that drive the distribution of species, of which prey distributions are likely important, and vary between populations and study areas. JWS are believed to mainly consume benthic elasmobranchs such as sting rays, which have reduced shifts in distribution throughout the year [66,67]. Yet tracking data from multiple populations have now shown large seasonal movements [36,65]. This study identified temperature as the predominant predictor of shifts in distribution; temperature should be a relatively robust predictor between locations as individuals' physiology is directly impacted by this variable.

Using this simple model we show general agreement with known JWS distributions suggesting that these variables have predictive importance to the species. This is important as even simple models from small sample sizes can be of utility when identifying sites for further surveying effort [68] and by incorporating the likely distribution of individuals into population models, better estimates of population size can be determined [69]. Using these simple predictive models allows researchers to more specifically test hypothesis and identify commonalities or divergent properties between populations potentially driving population specific demographics.

## Supporting information

S1 Fig Interpolated v. standardized positions. Linearly interpolated daily positions (A) and daily standardized positions (B) overlaid on top of the bathymetry data for Southern California. During periods when individuals were not detected, interpolated positions were often further from land, and less characteristic of assumed behavior.
(TIFF)

S2 Fig Interpolated v. standardized habitat selection. When GLMs are run on both interpolated (blue) and daily standardized (red) positions there is a greater selection for habitats that are further from land (A). The increased probability for locations that are further from land can be attributed to unlikely interpolated paths. During movements, individuals are likely to remain near to the shoreline, however interpolated straight tracks do not follow the coastline. Additionally there is a slight selection for cooler habitats in the interpolated data (B). From acoustic and satellite data (Lowe un pub.), individuals are observed to make highly directed movements when they shift habitats. If this shift happens during a prolonged period when the individual was not detected, interpolated positions can lag behind the true location of the individual, placing it into cooler waters than it actually inhabited.
(TIFF)

S3 Fig Release locations and tracks. A) Release location (red circles) of each individual. B) All daily standardized locations, color coded by each individual.
(TIFF)

S1 File Environmental data. This is a description of the spatial environmental data used.
(DOCX)

S1 Data SPOT tag tracks for all sharks used in this study. (CSV)

S1 Table Tracking durations. This is a table of the each individuals size, capture data, number of days detected and deployment duration.
(XLSX)

## References

[1] Avgar T, Mosser A, Brown GS, Fryxell JM (2013) Environmental and individual drivers of animal movement patterns across a wide geographical gradient. Journal of Animal Ecology 82: 96–106. 10.1111/j.1365-2656.2012.02035.x 23020517

[2] Shepard EL, Wilson RP, Rees WG, Grundy E, Lambertucci SA, Vosper SB(2013) Energy landscapes shape animal movement ecology. The American Naturalist 182: 298–312. 10.1086/671257 23933722

[3] Levings SC (1983) Seasonal, Annual, and Among‐site Variation in the Ground Ant Community of a Deciduous Tropical Forest: Some Causes of Patchy Species Distributions. Ecological Monographs 53: 435–455.

[4] Bailey H, Benson SR, Shillinger GL, Bograd SJ, Dutton PH, Eckert SA, et al (2012) Identification of distinct movement patterns in Pacific leatherback turtle populations influenced by ocean conditions. Ecological Applications 22: 735–747. 22645807 10.1890/11-0633

[5] Nicolson C, Berman M, West C, Kofinas G, Griffith B, Russel D, et al (2013) Seasonal climate variation and caribou availability: modeling sequential movement using satellite-relocation data. Ecology and Society 18.

[6] Dunn DC, Boustany AM, Halpin PN (2011) Spatio‐temporal management of fisheries to reduce by‐catch and increase fishing selectivity. Fish and Fisheries 12: 110–119.

[7] Žydelis R, Lewison RL, Shaffer SA, Moore JE, Boustany AM, Roberts JJ, et al (2011) Dynamic habitat models: using telemetry data to project fisheries bycatch. Proceedings of the Royal Society of London B: Biological Sciences 278: 3191–3200. 10.1098/rspb.2011.0330 PMC3169031 21429921

[8] Cosandey-Godin A, Krainski ET, Worm B, Flemming JM (2014) Applying Bayesian spatiotemporal models to fisheries bycatch in the Canadian Arctic. Canadian Journal of Fisheries and Aquatic Sciences 72: 186–197.

[9] Roe JH, Morreale SJ, Paladino FV, Shillinger GL, Benson SR, Eckert SA, et al (2014) Predicting bycatch hotspots for endangered leatherback turtles on longlines in the Pacific Ocean. Proceedings of the Royal Society of London B: Biological Sciences 281: 20132559. 10.1098/rspb.2013.2559 PMC3896015 24403331

[10] Hazen EL, Carlisle AB, Wilson SG, Ganong JE, Castleton MR, Schallert RJ, et al (2016) Quantifying overlap between the Deepwater Horizon oil spill and predicted bluefin tuna spawning habitat in the Gulf of Mexico. Scientific Reports 6. 10.1038/srep33824 PMC5031980 27654709

[11] Hahlbeck N, Scales KL, Dewar H, Maxwell SM, Bograd SJ, Hazen EL(2017) Oceanographic determinants of ocean sunfish (Mola mola) and bluefin tuna (Thunnus orientalis) bycatch patterns in the California large mesh drift gillnet fishery. Fisheries Research 191: 154–163.

[12] Richards RA (1992) Habitat selection and predator avoidance: ontogenetic shifts in habitat use by the Jonah crab *Cancer borealis* (Stimpson). Journal of Experimental Marine Biology and Ecology 156: 187–197.

[13] Dahlgren CP, Eggleston DB (2000) Ecological processes underlying ontogenetic habitat shifts in a coral reef fish. Ecology 81: 2227–2240.

[14] Hofmann N, Fischer P (2002) Temperature preferences and critical thermal limits of burbot: implications for habitat selection and ontogenetic habitat shift. Transactions of the American Fisheries Society 131: 1164–1172.

[15] Crouse DT, Crowder LB, Caswell H (1987) A stage-based population model for loggerhead sea turtles and implications for conservation. Ecology 68: 1412–1423.

[16] Miller TJ, Crowder LB, Rice JA, Marschall EA (1988) Larval size and recruitment mechanisms in fishes: toward a conceptual framework. Canadian Journal of Fisheries and Aquatic Sciences 45: 1657–1670.

[17] Laurenson MK (1995) Implications of high offspring mortality for cheetah population dynamics. Serengeti II: dynamics, management and conservation of an ecosystem 385: 400.

[18] Gosselin LA, Qian P-Y (1997) Juvenile mortality in benthic marine invertebrates. Marine Ecology Progress Series 146: 265–282.

[19] Sogard SM (1997) Size-selective mortality in the juvenile stage of teleost fishes: a review. Bulletin of marine science 60: 1129–1157.

[20] Fergusson I, Compagno LJV, Marks M (2009) Carcharodon carcharias. The IUCN Red List of Threatened Species 2009.

[21] Cliff G, Dudley S, Davis B (1989) Sharks caught in the protective gill nets off Natal, South Africa. 2. The great white shark *Carcharodon carcharias* (Linnaeus). South African Journal of Marine Science 8: 131–144.

[22] Lowe CG, Blasius ME, Jarvis ET, Mason TJ, Goodmanlowe GD, O'Sullivan JB (2012) Historic fishery interactions with white sharks in the Southern California Bight. Global Perspectives on the Biology and Life History of the White Shark’(Ed Domeier ML) pp: 169–186.

[23] Werry JM, Bruce BD, Sumpton W, Reid D, Mayer DG (2012) Beach areas used by juvenile white sharks, *Carcharodon carcharias*, in eastern Australia. Global Perspectives on the Biology and Life History of the White Shark: 271.

[24] Curtis TH, McCandless CT, Carlson JK, Skomal GB, Kohler NE, Natanson LJ, et al (2014) Seasonal distribution and historic trends in abundance of white sharks, *Carcharodon carcharias*, in the western North Atlantic Ocean. PloS one 9: e99240 10.1371/journal.pone.0099240 24918579 PMC4053410

[25] Ezcurra JM, Lowe CG, Mollet HF, Ferry LA, O’Sullivan JB (2012) Captive feeding and growth of young-of-the-year white sharks, Carcharodon carcharias, at the Monterey Bay Aquarium. Global perspectives on the biology and life history of the white shark: 1.

[26] Ezcurra JM, Lowe CG, Mollet HF, Ferry LA, O’Sullivan JB (2012) Oxygen consumption rate of young-of-the-year white sharks, Carcharodon carcharias, during transport to the Monterey Bay Aquarium. Global Perspectives on the Biology and Life History of the White Shark: 17–26.

[27] Weng KC, O’Sullivan JB, Lowe CG, Winkler CE, Blasius ME, Loke-Smith KA, et al (2012) Release of Juvenile White Sharks from. Global Perspectives on the Biology and Life History of the White Shark: 419.

[28] Weng KC, O’Sullivan JB, Lowe CG, Winkler CE, Dewar H, Block BA(2007) Movements, behavior and habitat preferences of juvenile white sharks Carcharodon carcharias in the eastern Pacific. Marine Ecology Progress Series 338: 211–224.

[29] Costa DP, Robinson PW, Arnould JP, Harrison A-L, Simmons SE, Hassrick JL, et al (2010) Accuracy of ARGOS locations of pinnipeds at-sea estimated using Fastloc GPS. PloS one 5: e8677 10.1371/journal.pone.0008677 20090942 PMC2806907

[30] Barbet‐Massin M, Jiguet F, Albert CH, Thuiller W (2012) Selecting pseudo‐absences for species distribution models: how, where and how many? Methods in Ecology and Evolution 3: 327–338.

[31] Burnham KP, Anderson DR, Huyvaert KP (2011) AIC model selection and multimodel inference in behavioral ecology: some background, observations, and comparisons. Behavioral Ecology and Sociobiology 65: 23–35.

[32] Zhang Y, Wallace JM, Battisti DS (1997) ENSO-like interdecadal variability: 1900–93. Journal of climate 10: 1004–1020.

[33] Lyons K, Jarvis ET, Jorgensen SJ, Weng K, O'Sullivan J, Winkler C, et al (2013) The degree and result of gillnet fishery interactions with juvenile white sharks in southern California assessed by fishery-independent and-dependent methods. Fisheries Research 147: 370–380.

[34] Bates D, Mächler M, Bolker BM, Walker SC (2015) Fitting linear mixed-effects models using lme4. Journal of statistical software 67.

[35] Domeier ML (2012) A new life-history hypothesis for white sharks, Carcharodon carcharias, in the Northeastern Pacific. Global Perspectives on the Biology and Life History of the White Shark: 199–224.

[36] Bruce BD, Bradford RW (2012) Habitat use and spatial dynamics of juvenile white sharks, *Carcharodon carcharias*, in eastern Australia. Global Perspectives on the Biology and Life History of the White Shark: 225–254.

[37] Hussey NE, McCann HM, Cliff G, Dudley SF, Wintner SP, Fisk AT (2012) Size-based analysis of diet and trophic position of the white shark (*Carcharodon carcharias*) in South African waters. Global Perspectives on the Biology and Life History of the White Shark’(Ed Domeier ML) pp: 27–49.

[38] Benson JF, Jorgensen SJ, O'Sullivan JB, Winkler C, White CF, Garcia-Rodriguez E, et al (2018) Juvenile survival, competing risks, and spatial variation in mortality risk of a marine apex predator. Journal of Applied Ecology.

[39] Dunham AE, Grant BW, Overall KL (1989) Interfaces between biophysical and physiological ecology and the population ecology of terrestrial vertebrate ectotherms. Physiological Zoology: 335–355.

[40] Huey RB (1991) Physiological consequences of habitat selection. American Naturalist: S91–S115.

[41] Gillooly JF, Brown JH, West GB, Savage VM, Charnov EL (2001) Effects of size and temperature on metabolic rate. science 293: 2248–2251. 10.1126/science.1061967 11567137

[42] Angilletta MJ (2009) Thermal adaptation: a theoretical and empirical synthesis: Oxford University Press.

[43] Angilletta MJ, Steury TD, Sears MW (2004) Temperature, growth rate, and body size in ectotherms: fitting pieces of a life-history puzzle. Integrative and Comparative Biology 44: 498–509. 10.1093/icb/44.6.498 21676736

[44] Di Santo V, Bennett W (2011) Is post‐feeding thermotaxis advantageous in elasmobranch fishes? Journal of Fish Biology 78: 195–207. 10.1111/j.1095-8649.2010.02853.x 21235555

[45] Goldman KJ (1997) Regulation of body temperature in the white shark, Carcharodon carcharias. Journal of Comparative Physiology B: Biochemical, Systemic, and Environmental Physiology 167: 423–429.

[46] Logan R, White CF, Winkler C, Jorgensen S, O’Sullivan J, Lowe CG, et al (2018) An evaluation of body condition and morphometric relationships within southern California juvenile white sharks Carcharodon carcharias. Journal of Fish Biology. 10.1111/jfb.13785 30141191

[47] Holland KN, Sibert JR (1994) Physiological thermoregulation in bigeye tuna, Thunnus obesus. Environmental Biology of Fishes 40: 319–327.

[48] Kitagawa T, Nakata H, Kimura S, Tsuji S (2001) Thermoconservation mechanisms inferred from peritoneal cavity temperature in free-swimming Pacific bluefin tuna Thunnus thynnus orientalis. Marine Ecology Progress Series 220: 253–263.

[49] Thums M, Meekan M, Stevens J, Wilson S, Polovina J (2012) Evidence for behavioural thermoregulation by the world's largest fish. Journal of The Royal Society Interface: rsif20120477. 10.1098/rsif.2012.0477 PMC3565775 23075547

[50] Nakamura I, Goto Y, Sato K (2015) Ocean sunfish rewarm at the surface after deep excursions to forage for siphonophores. Journal of Animal Ecology 84: 590–603. 10.1111/1365-2656.12346 25643743

[51] Andrzejaczek S, Gleiss AC, Jordan LK, Pattiaratchi CB, Howey LA, Brooks EJ, et al (2018) Temperature and the vertical movements of oceanic whitetip sharks, Carcharhinus longimanus. Scientific reports 8: 8351 10.1038/s41598-018-26485-3 29844605 PMC5974137

[52] Campana SE, Dorey A, Fowler M, Joyce W, Wang Z, Wright D, et al (2011) Migration pathways, behavioural thermoregulation and overwintering grounds of blue sharks in the Northwest Atlantic. PloS one 6: e16854 10.1371/journal.pone.0016854 21373198 PMC3044145

[53] Campana SE, Joyce W, Fowler M (2010) Subtropical pupping ground for a cold-water shark. Canadian Journal of Fisheries and Aquatic Sciences 67: 769–773.

[54] Santana-Morales O, Sosa-Nishizaki O, Escobedo-Olvera M, Oñate-González E, O'Sullivan J, Cartamil D (2012) Incidental catch and ecological observations of juvenile white sharks, *Carcharodon carcharias*, in western Baja California, Mexico: Conservation implications. Global perspectives on the biology and life history of the white shark: 187–198.

[55] Oñate-González EC, Sosa-Nishizaki O, Herzka SZ, Lowe CG, Lyons K, Stanta-Morales O, et al (2017) Importance of Bahia Sebastian Vizcaino as a nursery area for white sharks (Carcharodon carcharias) in the Northeastern Pacific: A fishery dependent analysis. Fisheries Research 188: 125–137.

[56] Murray KT (2004) Magnitude and distribution of sea turtle bycatch in the sea scallop (Placopecten magellanicus) dredge fishery in two areas of the northwestern Atlantic Ocean, 2001–2002. Fishery Bulletin 102: 671–681.

[57] Hobday A, Hartmann K (2006) Near real‐time spatial management based on habitat predictions for a longline bycatch species. Fisheries Management and Ecology 13: 365–380.

[58] Galván-Magaña F, Hoyos-Padilla EM, Navarro-Serment CJ, Márquez-Farías F (2010) Records of white shark, *Carcharodon carcharias*, in the Gulf of California, Mexico. Marine Biodiversity Records 3: e111.

[59] Ramirez-Amaro SR, Cartamil D, Galvan-Magaña F, Gonzalez-Barba G, Graham JB, Carrera-Fernandez M, et al (2013) The artisanal elasmobranch fishery of the Pacific coast of Baja California Sur, Mexico, management implications. Scientia Marina 77: 473–487.

[60] Hayward TL (1993) Preliminary observations of the 1991–1992 El Niño in the California Current. California Cooperative Oceanic Fisheries Investigations Reports 34: 21–29.

[61] Norton JG (1999) Apparent habitat extensions of dolphinfish (*Coryphaena hippurus*) in response to climate transients in the California Current. Scientia Marina 63: 261–266.

[62] Lea RN, Rosenblatt RH (2000) Observations on fishes associated with the 1997–98 El Niño off California. Reports of California Cooperative Oceanic Fisheries Investigations 41: 117–129.

[63] Skubel R, Kirtman B, Fallows C, Hammerschlag N (2018) Patterns of long-term climate variability and predation rates by a marine apex predator, the white shark Carcharodon carcharias. Marine Ecology Progress Series 587: 129–139.

[64] Kabasakal H (2014) The status of the great white shark (*Carcharodon carcharias*) in Turkey's waters. Marine Biodiversity Records 7: e109.

[65] Curtis TH, Metzger G, Fischer C, McBride B, McCallister M, Winn LJ, et al (2018) First insights into the movements of young-of-the-year white sharks (Carcharodon carcharias) in the western North Atlantic Ocean. Scientific reports 8: 10794 10.1038/s41598-018-29180-5 30018411 PMC6050330

[66] Vaudo J, Lowe CG (2006) Movement patterns of the round stingray Urobatis halleri (Cooper) near a thermal outfall. Journal of Fish Biology 68: 1756–1766.

[67] Farrugia TJ, Espinoza M, Lowe CG (2011) Abundance, habitat use and movement patterns of the shovelnose guitarfish (Rhinobatos productus) in a restored southern California estuary. Marine and Freshwater Research 62: 648–657.

[68] Baldwin RA (2009) Use of maximum entropy modeling in wildlife research. Entropy 11: 854–866.

[69] Hillary R, Bravington M, Patterson T, Grewe P, Bradford R, Feutry P, et al (2018) Genetic relatedness reveals total population size of white sharks in eastern Australia and New Zealand. Scientific reports 8: 2661 10.1038/s41598-018-20593-w 29422513 PMC5805677

