# Supplementary figures and images for: Quantifying habitat selection and variability in habitat suitability for juvenile white sharks
Source: PLoS One. 2019 May 8;14(5):e0214642. doi: 10.1371/journal.pone.0214642 (PMC6505937; doi:10.1371/journal.pone.0214642)

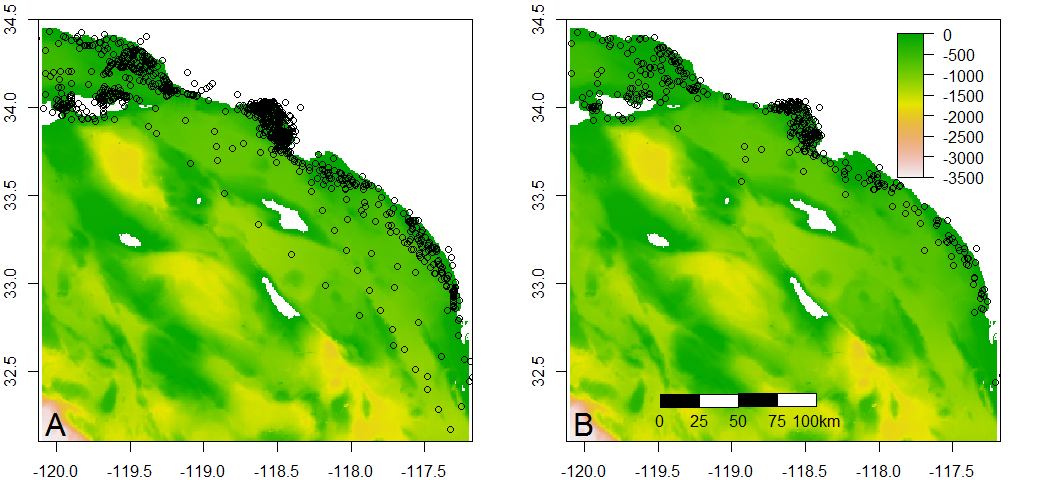

Supplement: S1 Fig — Linearly interpolated daily positions (A) and daily standardized positions (B) overlaid on top of the bathymetry data for Southern California. During periods when individuals were not detected, interpolated positions were often further from land, and less characteristic of assumed behavior. (TIFF) [file pone.0214642.s001.tiff]

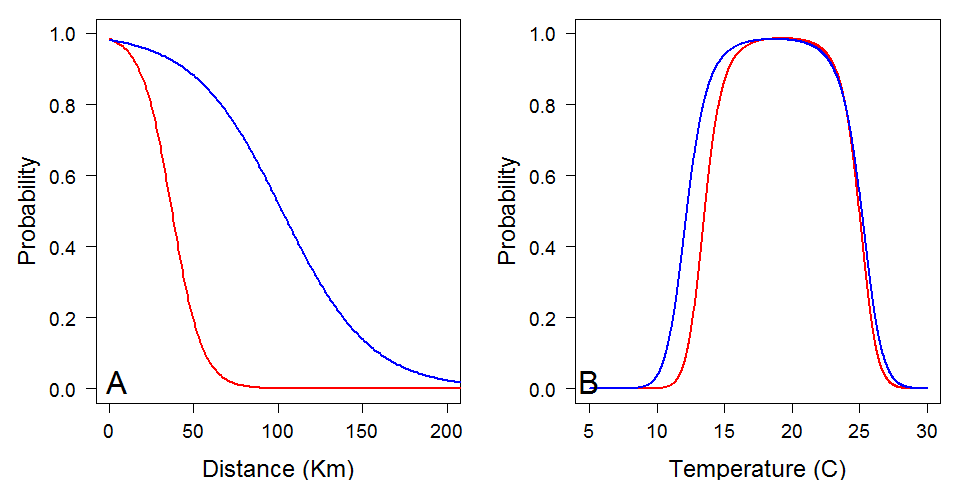

Supplement: S2 Fig — When GLMs are run on both interpolated (blue) and daily standardized (red) positions there is a greater selection for habitats that are further from land (A). The increased probability for locations that are further from land can be attributed to unlikely interpolated paths. During movements, individuals are likely to remain near to the shoreline, however interpolated straight tracks do not follow the coastline. Additionally there is a slight selection for cooler habitats in the interpolated data (B). From acoustic and satellite data (Lowe un pub.), individuals are observed to make highly directed movements when they shift habitats. If this shift happens during a prolonged period when the individual was not detected, interpolated positions can lag behind the true location of the individual, placing it into cooler waters than it actually inhabited. (TIFF) [file pone.0214642.s002.tiff]

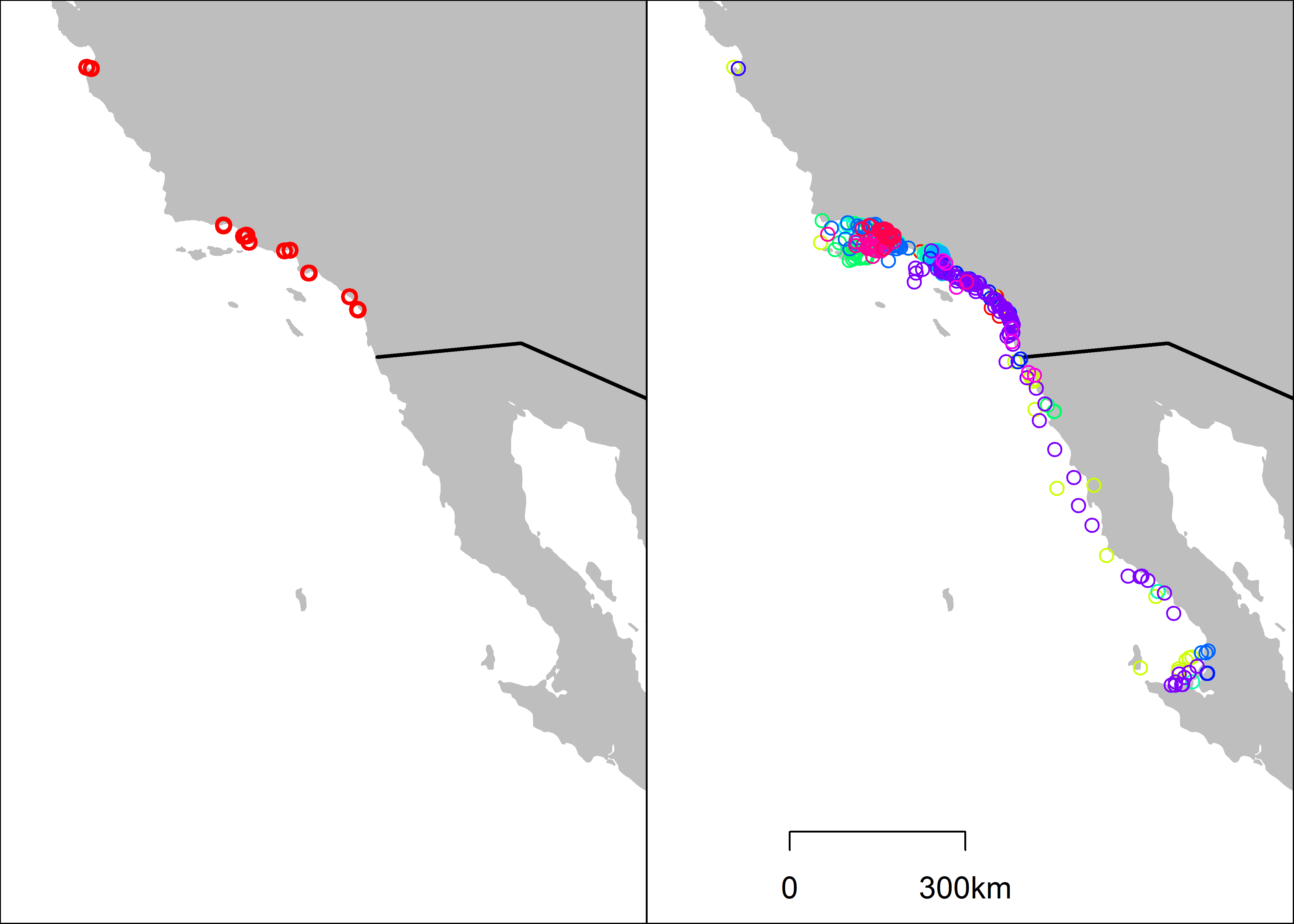

Supplement: S3 Fig — A) Release location (red circles) of each individual. B) All daily standardized locations, color coded by each individual. (TIFF) [file pone.0214642.s003.tiff]
