# Supplementary material for: Quantifying habitat selection and variability in habitat suitability for juvenile white sharks
Source: PLoS One. 2019 May 8;14(5):e0214642. doi: 10.1371/journal.pone.0214642 (PMC6505937; doi:10.1371/journal.pone.0214642)
Supplement: S1 File — This is a description of the spatial environmental data used. (DOCX) [file pone.0214642.s004.docx]

**Supporting Information 1 : Environmental Data**

**Static Habitat Maps**

Depth data was from the General Bathymetric Chart of the Oceans (British Oceanographic Data Centre) at a resolution of 30 arc seconds. In order to represent the complexity of the bottom and to identify areas of rapid change in bottom height (e.g., canyons, sea mounts, continental slope) a focal analysis was conducted to calculate depth gradient which is the range in depth over a 9 km^2^ focal window. For every location in the study area the distance to the closest land was calculated using the distance function in the r package *raster.*

**Dynamic Habitat Maps**

Global sea surface temperature maps were generated from the Multi-Scale Ultra-high Resolution Sea Surface Temperature (MUR) data provided by the Jet Propulsion laboratory (JPL). This dataset provides daily global SST maps from June 2002 through present, at a resolution of 0.011 degrees. This is a dataset based on post-processed and blended sea surface temperature data provided by multiple sources in order to interpolate across spatial scales and time in order to predict SST through cloud cover (Chao et al., 2009). SST gradients were calculated in order to identify SST thermal fronts. SST gradients were also a daily focal analysis that measured the range of temperatures within a 9 km^2^ window.

Global sea surface chlorophyll (Chl *a*) dataset was collected from the Ocean Color group using data collected from the MODIS Satellite. These data are collected at a 4 km resolution and was averaged weekly from June 2006 through December 2015 to account for periods when sea surface chlorophyll could not be measured due to daily cloud cover, and thus minimize gaps in coverage. In addition, sea surface chlorophyll is slower to respond to changing conditions and thus weekly data might be more representative. Similar to temperature and depth, the complexity in the spatial rate of change of chlorophyll could be an environmental characteristic that individuals were selecting. Thus, Chl *a* gradient was calculated as the range in chlorophyll values across a 9 km^2^ focal window.

Each dataset used represented one of the finest spatial and temporal resolutions available on a global-level, which allowed the same environmental predictors to be used in both regional and global analysis. However, environmental datasets were originally collected at different spatial resolutions. In order to run models, all maps spatial resolutions were standardized by resampling to be at the same spatial resolution of 0.01 degrees.
